# Auditory Time-Frequency Masking for Spectrally and Temporally Maximally-Compact Stimuli

**DOI:** 10.1371/journal.pone.0166937

**Published:** 2016-11-22

**Authors:** Thibaud Necciari, Bernhard Laback, Sophie Savel, Sølvi Ystad, Peter Balazs, Sabine Meunier, Richard Kronland-Martinet

**Affiliations:** 1 Acoustics Research Institute, Austrian Academy of Sciences, Vienna, Austria; 2 Laboratoire de Mécanique et d’Acoustique, CNRS UPR 7051, Equipe Sons, Aix-Marseille Université, Centrale Marseille, Marseille, France; Johns Hopkins University, UNITED STATES

## Abstract

Many audio applications perform perception-based time-frequency (TF) analysis by decomposing sounds into a set of functions with good TF localization (i.e. with a small essential support in the TF domain) using TF transforms and applying psychoacoustic models of auditory masking to the transform coefficients. To accurately predict masking interactions between coefficients, the TF properties of the model should match those of the transform. This involves having masking data for stimuli with good TF localization. However, little is known about TF masking for mathematically well-localized signals. Most existing masking studies used stimuli that are broad in time and/or frequency and few studies involved TF conditions. Consequently, the present study had two goals. The first was to collect TF masking data for well-localized stimuli in humans. Masker and target were 10-ms Gaussian-shaped sinusoids with a bandwidth of approximately one critical band. The overall pattern of results is qualitatively similar to existing data for long maskers. To facilitate implementation in audio processing algorithms, a dataset provides the measured TF masking function. The second goal was to assess the potential effect of auditory efferents on TF masking using a modeling approach. The temporal window model of masking was used to predict present and existing data in two configurations: (1) with standard model parameters (i.e. without efferents), (2) with cochlear gain reduction to simulate the activation of efferents. The ability of the model to predict the present data was quite good with the standard configuration but highly degraded with gain reduction. Conversely, the ability of the model to predict existing data for long maskers was better with than without gain reduction. Overall, the model predictions suggest that TF masking can be affected by efferent (or other) effects that reduce cochlear gain. Such effects were avoided in the experiment of this study by using maximally-compact stimuli.

## Introduction

It is of great interest in audio applications to take human auditory perception into account in the signal processing chain. This generally consists in performing a perceptually motivated time-frequency (TF) analysis of the signal. TF analysis is the domain of transforms that allow decomposing any signal into a set of elementary functions or “atoms” whose TF localization (i.e. their essential support in the TF domain) determines the resolution of the transform. The result of a signal decomposition is a set of TF coefficients that quantifies the degree of similarity between the analyzed signal and the elementary atoms. Displaying the magnitude of these coefficients leads to a so-called TF representation of the signal. To obtain a fine resolution in both time and frequency, atoms with a good TF localization are generally chosen (e.g. [1, 2]). To obtain a perceptually motivated TF analysis, one can choose a set of atoms whose duration and bandwidth approximate the time and frequency resolution of the human auditory system (see e.g. [3] for a linear or [4] for a nonlinear approach) and/or apply a psychoacoustic model of auditory masking to the coefficients of the transform. A typical example is perceptual audio coding. To reduce the digital size of audio files, audio codecs like mp3 decompose sounds into TF segments (ideally a transform approximating the auditory frequency resolution is used like in [5]) and then apply a masking model to reduce the bit rates in these segments (e.g. [6, 7]). Similarly, sparsity-based approaches combine TF decompositions and masking models to reduce the amount of nonzero TF coefficients [8, 9]. Audio restoration techniques (e.g., [10, 11]) also combine TF transforms and masking models to retrieve only the perceptually relevant information. As a last example, source separation algorithms estimate binary masks to weight the TF coefficients of sound mixtures based on auditory masking in order to separate the signal(s) of interest [12, 13].

In summary, many applications rely on psychoacoustic masking models to extract and process the perceptually most relevant information in the TF domain. From a signal processing viewpoint, the accurate prediction of masking interactions between TF coefficients requires masking data for stimuli with a good TF localization. However, little is known about the TF spread of masking for such well-localized signals. For that reason, most masking models used in applications were developed based on existing masking data that are largely confined to long and/or broadband stimuli. In order to fill this gap, the present study reports experimental data on TF masking for Gabor atoms, namely 10-ms Gaussian-shaped sinusoids, that are well localized in the TF plane and approximate the auditory frequency resolution around 4 kHz. A modeling approach is also presented with the aim to assess the potential effect of auditory efferents on the spread of TF masking.

Auditory masking has been the focus of many psychoacoustic studies over the last decades (for a review see e.g. [14]). Auditory masking refers to the degradation in the audibility of a sound, the “target”, by the presence of another sound, the “masker”. This effect is quantified by measuring the detection threshold of the target in the presence and absence of the masker. The difference in thresholds (in dB) then corresponds to the “amount of masking”. Masking has been extensively investigated with simultaneous and non-simultaneous presentation of masker and target. To describe the various settings in which masking can be measured, we introduce the following notations: *f*_*i*_, *D*_*i*_, and *L*_*i*_ that respectively correspond to the frequency, duration, and sound pressure level (SPL) of masker (*i* = *M*) or target (*i* = *T*). The frequency shift between masker and target is Δ*F* = *f*_*T*_ − *f*_*M*_ and the time shift Δ*T* is defined as the delay between masker onset and target onset.

In simultaneous masking, masker and target overlap in time (i.e. 0 ≤ Δ*T* < *D*_*M*_) and the amount of masking is measured for various Δ*F* s, resulting in the spectral masking function (e.g. [15–17]). In non-simultaneous masking, Δ*F* is most often zero and Δ*T* is varied, resulting in the temporal masking function (e.g. [18–20]). To reduce confusion effects related to the difficulty of distinguishing the target from the masker [21], most masking studies involve masker and target with different spectral and temporal properties (e.g. broadband masker vs. narrowband target and/or *D*_*M*_ > *D*_*T*_), in other words signals with poor localization in the TF plane. Nonetheless, the results of these studies were used to develop the models of spectral *or* temporal masking that are implemented in audio applications, like the ones mentioned above, to predict masking in the TF plane. A few algorithms, however, exploit both spectral and temporal masking [9, 22–24]. The predictions of TF masking from those algorithms are based on a simple superposition of spectral and temporal masking functions. However, the linear combination of temporal and spectral masking effects to predict TF masking has been shown to be clearly inconsistent with experimental data [16, 25].

**Fig 1:**
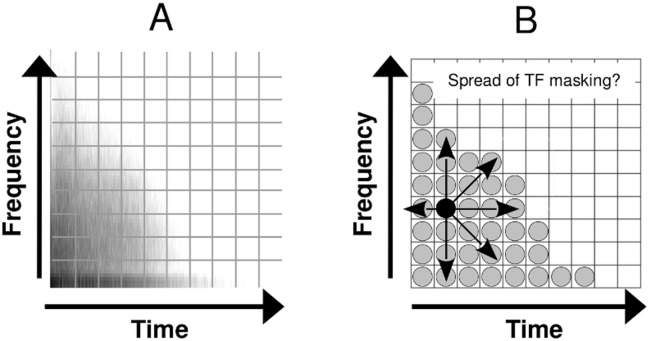
A. Time-frequency representation (amplitude of the Gabor transform) of a snare drum sound. B. Schematic decomposition of this signal into elementary Gabor atoms well localized in the TF plane. Arrows in B symbolize the spread of masking produced by a single “masker” atom highlighted in black.

Among the wide variety of TF analysis methods available, the short-time Fourier (also named Gabor) [2] or wavelet [1] transforms are often applied to audio signals. In Fig 1, the TF representation (here, a Gabor transform) of a snare drum sound is represented (A) along with the schematic decomposition of this signal into elementary Gabor atoms that are well localized in the TF plane (B). The black atom represents one potential masker. The vertical, horizontal, and diagonal arrows symbolize the spread of frequency, temporal, and TF masking, respectively, produced by this masker. Knowing the spread of TF masking for well-localized atoms is crucial for the accurate prediction of TF masking in the TF representations of arbitrary sounds. The reason for this is twofold. First, TF masking data for well-localized signals allow the development of a masking model that matches the basic property of TF transforms, namely the decomposition into well-localized atoms. Second, well-localized signals allow more flexibility for masking measurements in the TF plane compared to long and/or broadband signals. Using a long and narrowband masker, for instance, would constrain the sampling of the masking function along the time axis.

The temporal masking function measured for a given masker with fixed *f*_*M*_ and *L*_*M*_ and Δ*F* = 0 (e.g. the atom highlighted in black in Fig 1B) can be used as an indicator of the temporal spread of masking produced by this masker (consider the horizontal arrows as represented in Fig 1B). Similarly, the spectral masking function measured for a fixed masker with Δ*T* = 0 can be used as an indicator of its spectral spread of masking (resp. the vertical arrows in Fig 1B). A few studies using short sinusoidal maskers involved TF conditions, including temporal masking functions measured for Δ*F*≠ 0 (e.g. [26–29]) and spectral masking functions measured for Δ*T*≠ 0 [30]. Because these studies used a small set of Δ*F* s and Δ*T* s (at most two values tested), their results do not provide sufficient information on the spread of TF masking produced by such a well-localized masker.

Other studies using long (*D*_*M*_ ≥ 100 ms) sinusoidal maskers involved a larger set of TF conditions. These studies include measurements of psychophysical tuning curves (PTCs, e.g. [31–33]), filter functions [16, 34], and masking patterns for various Δ*T* s [18–20, 35, 36]. However, in the following we provide arguments for why these data may not suffice to estimate the TF spread of masking for the particular type of stimuli under consideration here, namely TF atoms well localized in the TF plane. PTCs and filter functions are measured for a fixed *f*_*T*_ and variable *f*_*M*_. To obtain a PTC, *L*_*T*_ is fixed at a low level and *L*_*M*_ at threshold (i.e. when the target is just audible) is measured for each *f*_*M*_. To obtain a filter function, *L*_*M*_ is fixed and *L*_*T*_ at threshold is measured for each *f*_*M*_. Because PTCs and filter functions are assumed to measure the response of a single auditory filter centered at *f*_*T*_, these two types of spectral masking functions are commonly used as estimates of auditory frequency resolution (e.g. [16, 30, 37]) and cannot be used as indicators of the spectral spread of masking produced by the masker. In contrast, masking patterns are measured for fixed *f*_*M*_ and *L*_*M*_ and variable *f*_*T*_ and *L*_*T*_. Masking patterns are assumed to measure the responses of different auditory filters, precisely those centered at the individual *f*_*T*_s, and therefore can be used as indicators of the spectral spread of masking produced by the masker [15, 17]. If the cochlea were a linear system, masking patterns could easily be derived from either PTCs or filter functions. The shape of a masking pattern should then resemble that of an inverted PTC or a mirrored filter function. However, it is well known that the cochlear response to sounds depends nonlinearly on sound level. Precisely, the cochlear response is compressive at mid-to-high levels and linear at low levels [38]. Consequently, a function measured by fixing *L*_*T*_ and varying *L*_*M*_ (e.g. a PTC) cannot be used to infer a function measured by fixing *L*_*M*_ and varying *L*_*T*_ (i.e. a masking pattern), unless cochlear compression is taken into account [33, 39]. Still, there are at least two reasons why estimating the TF spread of masking for a short and narrowband masker based on existing measurements of PTCs or filter functions for long and narrowband maskers is problematic. The first reason stems from the aforementioned assumption that masking patterns measure the responses of different auditory filters. Thus, not a single PTC or filter function but a *set* of functions is required to infer *one* masking pattern, namely the auditory filters’ responses for all *f*_*T*_s and a wide range of levels, which is typically not available. Second, there are indications that PTCs and filter functions measured for long maskers may differ from those measured for short maskers [30, 40, 41], but see [39] for alternative results.

One potential factor that may induce different masking patterns for very short (*D*_*M*_ ≤ 10 ms) maskers compared to the long maskers tested in the literature [18–20, 35, 36] could be the activation of a feedback loop in the auditory system, the medial olivocochlear reflex (MOCR). The MOCR controls the cochlear gain via efferent connections to the outer hair cells on the basilar membrane [42]. It is believed that activation of the MOCR can affect masking by reducing cochlear gain and, as a byproduct, by reducing frequency resolution and cochlear nonlinearity (e.g. [37, 41, 43, 44]). This reflex has a delay of about 20–25 ms between the onset of the stimulus and the onset of the gain reduction [42]. It follows that in masking conditions involving Δ*T* s greater than about 25 ms, which is inevitably the case in temporal masking when *D*_*M*_ > 25 ms, the cochlear response to the target is supposedly affected by the MOCR-induced gain reduction. This might not be true in conditions involving very short maskers and Δ*T* s. Accordingly, the results from some masking experiments suggest that the parameters *D*_*M*_ and Δ*T* as well as the presence of a “precursor” signal preceding the masker all affect the spread of spectral (e.g. [43–47]) and temporal masking (e.g. [27, 30, 40, 41]). Moreover, recent modeling work has shown that adding a gain reduction stage in current models of masking could qualitatively account for some of the changes in spectral and temporal masking associated with *D*_*M*_, Δ*T*, and the presence of a precursor [29, 37, 48, 49]. Based on these considerations we concluded that the spread of TF masking for a well-localized signal can hardly be derived from existing data and has therefore to be measured.

In this study, we collected masking data for 10-ms Gabor atoms to characterize TF masking for this particular class of stimuli that may be useful for audio signal processing applications. Gabor atoms have Gaussian shapes in both time and frequency and minimize the TF uncertainty principle [1, 2]. Additionally, it has been suggested that Gaussian-shaped stimuli with an appropriate support in the TF domain excite a limited number of hypothesized TF observation windows of the auditory system compared to broadband and/or long stimuli [50]. Using stimuli with a compact support in the time domain also has the potential advantage to avoid the influence of auditory efferents like the MOCR. We verified this using a modeling approach. Specifically, we tested the ability of a well-established model of masking that does *not* include MOCR effects, namely the temporal window model [51, 52], to predict the present data and existing TF masking data measured for long maskers. We hypothesized that the 10-ms Gabor atoms of interest, unlike long maskers previously tested, are too short to induce any MOCR-induced gain reduction during the presentation time of the target. For this reason, the temporal window model may be most appropriate to predict the TF spread of masking for such atoms. In contrast, a model including MOCR effects may be most appropriate to predict existing TF masking data. To test this hypothesis, the temporal window model was used in two configurations: without MOCR effect (i.e. using standard model parameters [52]) and with simulation of the MOCR effect (i.e. by adding a 15-dB cochlear gain reduction following the approach in [37, 48]). Our hypothesis was that the standard configuration (i.e. without MOCR) would better predict the present data than the MOCR configuration. Conversely, the MOCR configuration would better predict existing long-masker data than the standard configuration.

## Methods

### Listeners

Four normal-hearing listeners participated in the experiment. All had thresholds of 15 dB HL or lower for octave frequencies from 125 Hz to 8 kHz and had no indications of hearing disorders. Two of them (L2 and L3) were highly experienced in psychoacoustics tasks. All listeners were volunteer and gave informed consent before the experiment. They were informed on the procedure and were free to withdraw at any time. The consent was verbal because the study involved no procedures for which written consent is normally required: (1) the study presented no more than minimal risk to the listeners (headphone presentation at low SPLs), (2) the alteration of consent procedures would not have affected the listeners’ rights, (3) the listeners were not deprived of any financial benefits because there was no commercial exploitation of the data, (4) there was sufficient protection of listeners’ privacy; and (5) the confidentiality of the data was implicit as no personally identifiable information (such as names or addresses) of the listeners was collected. Further, the directors of the Laboratoire de Mécanique et d’Acoustique waived the need for approval by an ethical review board.

### Material

All stimuli were digitally generated at a 48-kHz sampling rate and a 24-bit resolution using a digital array processing card (Tucker-Davis Technologies, TDT, System III) piloted by a Delphi program running on a PC. Masker and target were computed in Delphi and routed to two different channels of the processor (TDT RP2.1) and two digital-to-analog converters (DAC). When a continuous noise was needed to mask cochlear distortion products (see below), a white noise was generated in real time, lowpass-filtered (TDT RP2.1), and presented through the masker’s channel. The outputs of the two DACs were attenuated (TDT PA5) and added (TDT SM3) before being passed to a headphone buffer (TDT HB7), and to the right ear-pad of a circumaural headphone (Sennheiser HD 545). The headphones were calibrated so that levels were considered as SPLs close to the eardrum. Listeners were tested individually in a double-walled sound-attenuated booth.

### Stimuli

Masker and target were Gaussian-shaped sinusoids (Gabor atoms) defined by

$$\begin{array}{c} s_{i,\tau }\left(t\right)=\sqrt{\Gamma }\sin(2\pi f_{i}\left(t-\tau \right)+\frac{\pi }{4})e^{-\pi {\left(\Gamma (t-\tau )\right)}^{2}},\;i=\left\{M,T\right\} \end{array}$$ (1)

where *f*_*i*_ is the carrier frequency, *τ* is a time delay, and Γ = *α*_*i*_ *f*_*i*_. For a given *f*_*i*_, the shape factor of the Gaussian window, *α*, enables control of the spectro-temporal width (i.e. the support in the TF domain) of *s*_*i*,*τ*_. In particular, the equivalent rectangular bandwidth of *s*_*i*,*τ*_, *ERB*_*s*_*i*__ = Γ. The value of *α* was chosen according to the study by van Schijndel *et al*. [50], who used similar Gaussian stimuli to measure spectro-temporal integration. They made the assumption that the auditory system performs a TF analysis through its own TF windows. They attempted to characterize the spectro-temporal width of these elementary TF observation windows by assessing just noticeable differences in intensity for *s*_*i*,*τ*_ with various *α*s. They found that for *f*_*i*_ = 4 kHz the bandwidth of *s*_*i*,*τ*_ with *α* = 0.15 approximates the auditory critical bandwidth.

The masker used here was defined by *s*_*M*,0_ with *f*_*M*_ = 4 kHz and the target by *s*_*T*,Δ*T*_ with *f*_*T*_ = *f*_*M*_ + Δ*F*. In order to keep *ERB*_*s*_*i*,*τ*__ constant we fixed Γ to 600 Hz (i.e. Γ = 0.15 × 4000). The reason for keeping Γ instead of *α* constant was twofold: maintain the spectro-temporal widths of *s*_*M*,0_ and *s*_*T*,*τ*_ constant and comply with a Gabor-type analysis where the TF resolution is fixed (see Fig 1). By introducing the *π*/4 phase shift, the energy of the signal is independent of *f*_*i*_ [50].

Because a Gaussian window has an infinite duration, a criterion had to be found to limit the signal *s*_*i*,*τ*_(*t*) by temporally windowing it while preserving the properties of the Gaussian. This was achieved using a numerical optimization procedure developed by Depalle and Hélie [53], who designed a family of finite-duration windows with no spectral sidelobes based on Gaussian functions. Specifically, a Gaussian window has no spectral sidelobes and quickly tends towards zero but is not time limited. Multiplying a Gaussian function g with any window *w* in the temporal domain results in the convolution product of the respective Fourier transforms of g and *w*. When the spectral width of the Gaussian is large enough, the shallow decay removes the sidelobes of *w* in the result of the convolution. In the present study, the bandwidth of the Gaussian window was fixed by Γ (see Eq (1)). The procedure therefore computed by dichotomy the shortest duration of *w* (corresponding to Γ) that avoided sidelobes. As a result, the Gabor atoms were windowed in the time domain using a Tuckey window with a duration of 9.6 ms (i.e. *D*_*M*_ = *D*_*T*_ = 9.6 ms). The main lobe of the resulting windowed Gaussian in the frequency domain emerged from the asymptotic level by about 220 dB. The SPL of the Gaussian atom was specified by measuring the SPL of a pure tone having the same frequency (*f*_*i*_) and maximum amplitude as the carrier of the Gaussian.

### Experimental Conditions

**Fig 2:**
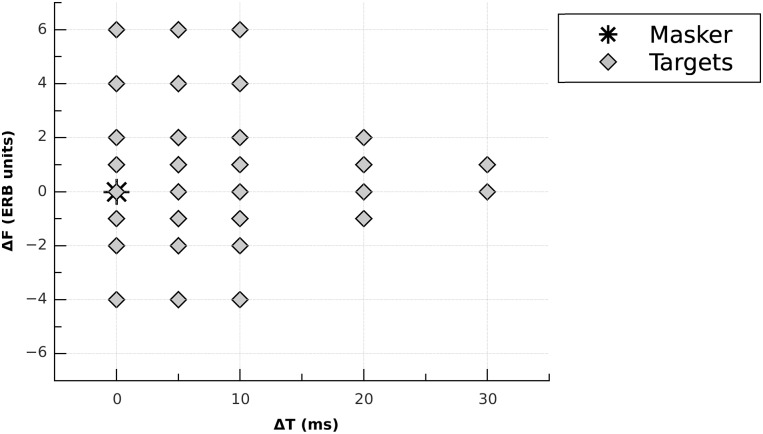
Experimental conditions summarized in the TF plane: set of Δ*F* s (in ERB units) measured for each Δ*T* (in ms). The black star indicates the position of the masker *s*_*M*,0_. Grey diamonds indicate the different positions of the target *s*_*T*,Δ*T*_ relative to the masker.

The masker had a fixed frequency *f*_*M*_ of 4 kHz and was presented at a sensation level of 60 dB (i.e. 60 dB above quiet threshold). The corresponding masker SPL *L*_*M*_ ranged between 81 and 86 dB across the listeners. Masking patterns were measured for eight Δ*F* s defined in the ERB scale [54] (-4, -2, -1, 0, +1, +2, +4, and +6 ERB units; *f*_*T*_s = 2521, 3181, 3568, 4000, 4480, 5015, 6274, and 7835 Hz, respectively) and five Δ*T* s (0, 5, 10, 20, and 30 ms). The range of Δ*F* s was chosen to avoid too large differences in the number of critical bands excited by the Gaussians. With “*ERB*_*f*_*T*__” defined as the ERB of the critical band centered at *f*_*T*_, the ratio *ERB*_*s*_*T*,Δ*T*__/*ERB*_*f*_*T*__ was limited to values between 0.5 and 2.0. To determine the amount of masking at each Δ*F*, the thresholds in quiet for the eight Gaussian targets were measured in a preliminary experiment. Values of Δ*T* < 0 were not tested because a pilot experiment indicated very little backward masking for such short stimuli. A pilot experiment indicated a very narrow spread of spectral masking for Δ*T* = 20 and 30 ms. Thus, only a subset of Δ*F* s was measured in these conditions. Overall, masked thresholds were measured for 30 out of 40 possible Δ*T* × Δ*F* combinations. These 30 conditions are represented in Fig 2.

When a combination of tones or complex sounds are processed by the ear, components that are not present in the acoustic signal can be heard. Such components are called distortion products and are thought to result from the nonlinear cochlear processing [38, 55]. In masking experiments, the detection of distortion products can help listeners solving the task and thus produce irregularities in the masking patterns, in particular when *f*_*T*_ is above *f*_*M*_ [14, 17]. To prevent cochlear distortion products from being detected, a continuous background signal was added in the present study. This signal was a low-pass filtered (-96 dB/octave) white noise whose cutoff frequency and level were determined so as to mask the most prominent distortion product, namely the cubic difference tone (CDT) [55]. The frequency of the CDT is *f*_*CDT*_ = 2*f*_*M*_ − *f*_*T*_. According to the power spectrum model of masking [54], which assumes that the detection of a sinusoidal component in a narrowband noise depends on a critical target-to-noise power ratio within an ERB, the cutoff frequency of the noise was chosen as the upper edge of the ERB centered at *f*_*CDT*_. The highest level of the CDT was estimated at about 25 dB below *L*_*M*_ [55]. Hence, the noise level was adjusted so as to totally mask a Gaussian *s*_*i*,0_(*t*) with *f*_*i*_ = *f*_*CDT*_ and *L*_*i*_ = *L*_*M*_ − 25. For all listeners, this was achieved using an overall noise level of 50 dB SPL. The lower- and higher-order distortion products (whose frequencies lie below *f*_*CDT*_) were presumably easily masked by the noise [55]. Although the effect of distortion products is usually ignored in forward masking studies, we used the background noise when *f*_*T*_ was above *f*_*M*_ because of the small Δ*T* s. We verified that the background noise had no influence on masker or target detection by measuring masking both with and without noise in the condition Δ*F* = 0, where no distortion product was generated. The results showed little or no effect of the background noise for all listeners and Δ*T* s (differences < 3 dB).

The whole set of conditions was split into two groups of Δ*F* s: measured with background noise (Δ*F* = 0, +1, +2, +4, and +6 ERB units) and measured without background noise (-4, -2, -1, and 0 ERB units). Then, experimental blocks were formed that contained the Δ*T* conditions for each Δ*F*. The order of blocks and groups was randomized across sessions. Within a session, the target frequency was fixed and Δ*T* was chosen randomly.

### Procedure

Thresholds were estimated using a three-interval, three-alternative forced-choice procedure with a 3-down-1-up criterion that estimates the 79.4%-correct point on the psychometric function [56]. The target was presented randomly in one of the three intervals. In absolute threshold measurements, the other two intervals were silent. In masked threshold measurements, all three intervals contained the masker. The listeners had to indicate which interval contained the target by pressing one of three buttons of a response box. Each 200-ms interval was visually indicated by lights on the response box, with a between-interval gap of 800 ms. Within each interval, the onset of the first Gaussian coincided with the onset of the interval. Visual feedback on the correctness of the response was provided at each trial. The starting level of the target was about 10–15 dB above the expected threshold, as determined by practice trials. The target level varied adaptively by initial steps of 5 dB and of 2 dB following the second reversal. Twelve reversals were obtained. The threshold estimate was the target level averaged across the last 10 reversals. A threshold estimate was discarded when the standard deviation across these 10 reversals exceeded 5 dB. Before data collection, practice series were performed for a number of conditions until the threshold estimates became stable. Then, two threshold estimates were obtained for each condition. If the standard deviation of these two estimates exceeded 3 dB, up to four additional estimates were completed. The final threshold was the average across all estimates obtained after practice series (maximum = 6). Listeners completed 1–2 sessions of 30 min per day for 10 to 15 non-consecutive days. The total testing time, including the measurement of absolute thresholds and practice series, was about 10 hours.

### Modeling

We based our analysis on a well-established model of masking and temporal resolution, the temporal window model [51, 57]. The general structure of the model consists of four stages: a middle-ear filter, a cochlear filter followed by a compressive function, a rectifier followed by a temporal integrator, and a decision device. The rationale for the model is that masking results from cochlear nonlinearities followed by a linear process, namely the temporal integrator. We used two different implementations of the original temporal window model proposed in [51]:

- Model 1: the dual-resonance nonlinear (DRNL) temporal window model [52].
- Model 2: the power-law temporal window model [37].

The only difference between these two implementations lies in the second stage, the cochlear model. Model 1 uses the DRNL filter bank proposed in [58]. Model 2 uses the peripheral processing stage of the power-law model proposed in [59, 60]. Both cochlear models have been shown to successfully predict physiological measurements of the cochlear responses to sounds. The parameters of these cochlear models were derived from physiological studies using anesthetized animals. Because anesthesia has been shown to significantly reduce efferent activity [61], both models are assumed to *exclude* efferent effects like the MOCR. Furthermore, the amount of cochlear compression in model 1 is consistent with recent estimates of cochlear compression in conditions *without* efferents (e.g. [28, 41, 62]). Nevertheless, the cochlear gain in these models can be reduced to simulate MOCR activation [37, 48]. Model 1 was successful at predicting various frequency-dependent nonlinearities in temporal and simultaneous masking such as the upward spread of forward masking and two-tone suppression [52, 63]. Model 2 was successful at qualitatively predicting PTCs measured in forward masking [37]. In other words, both models were already tested on TF conditions.

#### Procedure

To estimate thresholds, the masker alone and then the masker plus target signals were passed through the model. Detection was based on the ratio masker-plus-target/masker alone at the output of the model. This detection approach is similar to a two-alternative forced-choice task commonly used in masking studies [51]. The ratio masker-plus-target/masker alone for which the target is detected is defined as the model parameter *k*, in dB. To predict a single data point, the dependent variable (*L*_*T*_) was varied until the ratio masker-plus-target/masker alone equals a predefined *k*. This process was repeated for an entire data set. Note that *k* represents the detection efficiency, or sensitivity, of a listener and can be considered as a measure of Δ*L*. In the subsequent modeling, the only model parameter that was varied was *k*.

The simulation procedure included three steps. Step 1 consisted in finding the optimum value of *k* allowing to predict *L*_*T*_ in the condition Δ*T* = 0, Δ*F* = 0. This criterion was chosen because the Δ*T* = 0, Δ*F* = 0 condition in our study mimics an intensity discrimination task (see below), that is, a measure of Δ*L* like *k*. Step 2 consisted in using the *k* value found in step 1 and simulating masking patterns using the original model parameters (i.e. supposedly without any efferent effects like MOCR). Similarly, step 3 consisted in using the *k* value found in step 1 but applying a gain reduction of 15 dB to simulate the MOCR. The same approach was used in [48] and [37] to account for efferent effects in the DRNL filter and in the power-law model, respectively. The procedure was repeated for each model. Overall, a single value of *k* was used for the whole data set. Although the detection efficiency may vary between listeners, fixing *k* seems reasonable here because our goal was to simulate mean data.

#### Model Parameters

A Matlab implementation of the DRNL temporal window model provided by the first author in [52] was used. Noteworthy, the model version presented in [52] does not include the middle-ear filter. The middle-ear filter was added in [63] and was used in all simulations reported below. All model parameters (with the exception of *k*) were set at the original values indicated in [52] (see Table 1 therein). To obtain the power-law temporal window model, the DRNL filter was replaced by the peripheral processing stage (from the model’s input to the inner-hair-cell module’s output) of the power-law model described in [59, 64]. Because the power-law model features its own middle-ear filter, the middle-ear filter of the DRNL temporal window model was bypassed. While the authors in [37] used the cat version of the power-law model, we used the recent version of the power-law model [60] adapted for human hearing properties using the cochlear tuning from Glasberg and Moore (1990) [54]. We chose this tuning because the ERBs of our Gaussian stimuli were set according to the ERB scale in [54]. All other model stages remained unchanged and correspond to the model description in [37].

**Table 1:** RMS error (in dB) and squared Pearson’s correlation (*r*^2^) between data and simulations presented in Fig 5.

	Δ*T* = 0 ms		Δ*T* = 5 ms		Δ*T* = 10 ms		Δ*T* = 20 ms		total	
	error	*r*^2^	error	*r*^2^	error	*r*^2^	error	*r*^2^	error	*r*^2^
**model 1**										
standard	8.4	0.72	3.9	0.83	5.0	0.66	6.2	0.97	5.7	0.92
MOCR simulation	9.1	0.67	15.7	0.80	16.8	0.80	20.2	0.99	14.3	0.77
**model 2**										
standard	11.8	0.88	5.8	0.61	6.6	0.33	6.6	0.94	7.7	0.89
MOCR simulation	12.1	0.76	14.0	0.62	15.3	0.37	15.7	0.95	13.2	0.84

In both models and for each condition, the center time of the temporal window was always fixed and determined by the occurrence of the maximal rectifier output value of the masker-plus-target/masker alone ratio. Similarly, the center frequency of the cochlear filter was always fixed at the maximal frequency value of the masker-plus-target/masker alone ratio at the output of the temporal window. That is, the model always selected the “best” internal TF window to detect the target. (To find the best filter, a range of 10 filters below and 10 filters above *f*_*T*_ were simulated with center frequencies spaced in increments of 0.25 ERB units.) In other terms, we allowed for off-frequency listening, which happens when the target is detected using an auditory filter different from that centered at *f*_*T*_, and off-time listening (the analogy of off-frequency listening in the time domain) in the model. Accordingly, no stimulus to prevent off-frequency and off-time listening (e.g. a notched-noise like in [44]) was used in our experiment. To simulate the threshold in quiet, a constant was added to the output of the temporal window. This constant was equal to the minimum audible pressure (MAP) curve (precisely the ISO 226:2003 standard converted to MAP using the method from [65]) up-shifted in level by 13.29 dB, i.e. the difference in the mean threshold in quiet for the Gaussian and the MAP curve at 4 kHz.

## Results and Discussion

### Masking Data

**Fig 3:**
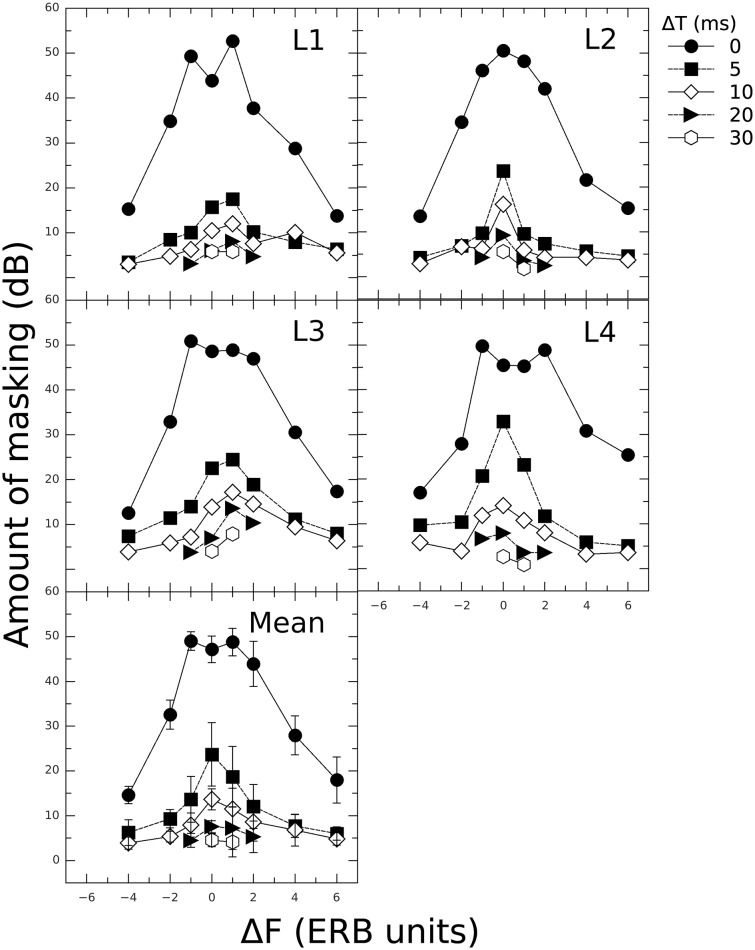
Experimental results plotted as masking patterns: Individual amounts of masking (in dB) as a function of Δ*F* (in ERB units) with Δ*T* as parameter. Note that for the conditions Δ*F* = 0 that were measured with and without background noise, the thresholds reported here correspond to the condition without noise. The bottom panel shows the mean data. Error bars indicate ±1 standard deviation of the mean data.

Individual and mean masking patterns are presented in Fig 3. For all Δ*F* s, the largest amount of masking was obtained in the simultaneous condition (Δ*T* = 0). In this condition, two listeners showed a dip (L1 and L4) and one a plateau (L3) instead of a peak at Δ*F* = 0. The Δ*F* = 0, Δ*T* = 0 condition represents a special case where masker and target were exactly the same stimuli presented synchronously. Thus, the listeners could only use the intensity increase in the interval containing the target as a cue. In other words, the listeners effectively performed an intensity discrimination task in this condition (e.g. [17]). The relative intensity increment Δ*I*/*I* in the interval containing both masker and target can be computed based on *L*_*M*_ and *L*_*T*_. Precisely, $\Delta I/I=\frac{I_{M+T}}{I_{M}}-1$ where *I*_*M*+*T*_ = 10^*L*_*M*+*T*_/10^ and *I*_*M*_ = 10^*L*_*M*_/10^. The logarithmic values of the intensity increment 10log(Δ*I*/*I*) for the condition Δ*F* = 0, Δ*T* = 0 are -4.7, -1.1, -2.1, and -3.9 dB for listeners L1-L4, respectively (mean = -2.9 dB). These values are similar to the average logarithmic intensity increment of -2.3 dB measured for 2-ms sinusoids and a baseline SPL of 85 dB (i.e. similar to *L*_*M*_ in the present study) in [66]. The presence of a peak in the masking pattern for listener L2 could be explained by poorer intensity discrimination performance of that listener compared to the other listeners. Moreover, note that the intensity increase in the target interval depends on the phase relationship between masker and target. In particular, when masker and target are presented with a 90-degree phase shift, the relative intensity increment is equal to the target-to-masker intensity ratio, i.e. Δ*I*/*I* = *I*_*T*_/*I*_*M*_ [67]. Using the Δ*I*/*I* values calculated above, it is thus possible to predict the values of *L*_*T*_ that would have been obtained if masker and target were presented with a 90-degree phase shift *and* an intensity discrimination cue was used in the present experiment. Precisely, *L*_*T*_ = *L*_*M*_ + 10 log(Δ*I*/*I*). These values of *L*_*T*_ are 79.2, 83.1, 79.6, and 79.28 dB for listeners L1-L4, respectively (mean = 80.3 dB). The corresponding amounts of masking are 55.3, 58.9, 57.9, and 56.2 dB (mean = 57 dB). For all listeners, these amounts of masking are much larger than those obtained for Δ*F* = -1 and +1 ERB unit. Thus, if we had used stimuli with a 90-degree phase shift, as done for instance in [17], the masking patterns would probably have shown a peak instead of a dip or plateau at Δ*F* = 0, Δ*T* = 0.

When Δ*T* increased, the dip/plateau observed at Δ*F* = 0 and Δ*T* = 0 disappeared. Masking dropped by 20–40 dB as Δ*T* increased to 5 ms for |Δ*F*| ≤ 2 ERB units. This drop was smaller for larger frequency shifts. For Δ*T* > 10 ms, masking was generally less than 10 dB for all Δ*F* s. Overall, this reflects a flattening of the patterns with increasing Δ*T*. To demonstrate this, the quality factors at the -3-dB bandwidth (*Q*_3_) were estimated for the patterns for Δ*T* = 0–10 ms. Estimates of *Q*_3_ were obtained by calculating the intersection of the lower (Δ*F* ≤ 0) and upper (Δ*F* ≥ 0) linear regression lines and dividing the frequency (in Hz) of the intersection point by the bandwidth (in Hz) 3 dB below the amount of masking at the intersection point. Values of *Q*_3_ were 9.8, 4.8, and 2.5 for Δ*T* = 0, 5, and 10 ms, respectively. Decreasing *Q*_3_ values indicate a broadening, or flattening, of the patterns with increasing Δ*T*.

**Fig 4:**
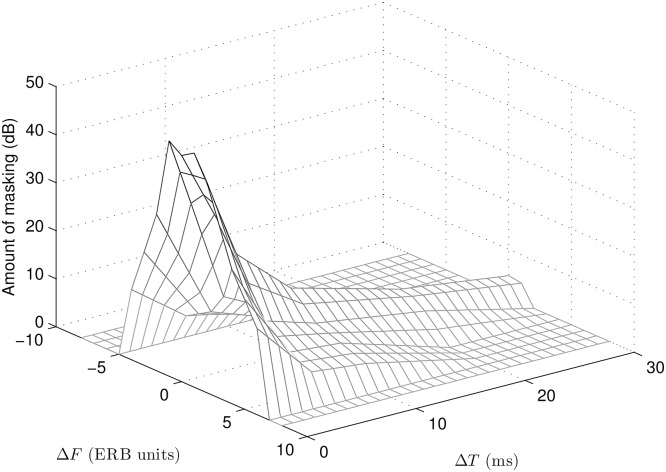
Mean amount of masking (in dB) as a function of Δ*T* (in ms) and Δ*F* (in ERB units). The spacing along the Δ*T* axis is 1 ms and the spacing along the Δ*F* axis is 1 ERB unit. The data for Δ*F* s above -4 and below +6 ERB units were interpolated based on a two-dimensional linear fit along the TF plane. For sampling points outside the range of measurements (i.e. for Δ*F* < -4 and Δ*F* > +6 ERB units), an extrapolation value of 0 dB was used. See the S1 Dataset for raw data.

The experimental results are summarized in the three-dimensional plot in Fig 4. To provide a smooth and “complete” representation of TF masking (i.e. one that reaches 0 dB of masking), the Δ*T* axis ranges from 0 to 30 ms using a regular spacing of 1 ms and the Δ*F* axis ranges from -8 to +8 ERB units using a regular spacing of 1 ERB unit. The data for Δ*F* s above -4 and below +6 ERB units were interpolated based on a two-dimensional linear fit along the TF plane. For sampling points outside the range of measurements, an extrapolation value of 0 dB was used. The function shown in Fig 4 represents the TF spread of masking produced by a Gaussian TF atom. To facilitate the implementation of this masking function in an audio processing algorithm, the data from Fig 4 are provided in S1 Dataset.

As mentioned above, a few studies measured masking patterns for various Δ*T* s [18–20, 35, 36]. These studies involved sinusoidal maskers (*f*_*M*_ = 1, 4, or 6 kHz) with *L*_*M*_s of 60–85 dB and *D*_*M*_s of 100–500 ms. Their results are qualitatively similar to ours in that masking for all Δ*F* s is greatest for the smallest Δ*T* tested and the patterns flatten with increasing Δ*T*. However, these studies reported forward masking for much larger Δ*T* s (≥ 100 ms) than the present study (≤ 20 ms). This is a consequence of the shorter *D*_*M*_ in the present study, consistent with results in [18, 27]. Because the present study involved Δ*T* s shorter than the MOCR onset delay (≈ 25 ms), our results were presumably not affected by any MOCR-induced gain reduction, unlike previous results. How the time course of MOCR-induced gain reduction affects the dependency of spectral masking decay on time (i.e. how masking patterns flatten as Δ*T* increases) is unclear. It is therefore not possible to determine whether our data with a very short masker can be derived from previous data with long maskers. It is possible, however, to predict the role of the MOCR on masking patterns using a masking model. This is considered in the next section.

Finally, note that the condition Δ*F* = 0, Δ*T* = 20 ms can be compared to one condition measured in [68] using identical Gaussian stimuli and procedures but different listeners and equipment. Specifically, the forward masker *M*_1_ in that study was presented 24 ms before the target at a sensation level of 59 dB, which corresponds to Δ*F* = 0, Δ*T* = 24 ms in the present setting. This masker produced an average amount of masking of 6.1 dB, which is consistent with the average amount of masking of 7.5 dB obtained here for Δ*F* = 0, Δ*T* = 20 ms. Although both studies share only one condition, this suggests that the overall amount of masking is consistent between the two groups of normal-hearing listeners.

### Model predictions

The main idea behind the present modeling attempt was twofold: to test the ability of the temporal window model to predict our data and to predict the effects of simulating MOCR-induced gain reduction (see Methods section for model description and fitting procedure). Our assumption was that simulating gain reduction is not required to predict our data due to the short masker duration *D*_*M*_ and time shifts Δ*T* s involved. Adding a gain reduction was mainly expected to result in higher predicted target SPL *L*_*T*_s than in the standard condition. Indeed, to compensate for the reduction in gain, higher *L*_*T*_s are in principle required at the model’s input to maintain the same value of *k* at the model’s output. The situation is not so simple in practice, since the ratio of masker-plus-target to masker alone at the output of the nonlinear cochlear filter highly depends on the relative positions of *L*_*M*_ and *L*_*T*_ on the compressive function [51, 57]. Thus, in conditions where masker and target are equally affected by the gain reduction, particularly for very small Δ*F* and Δ*T* where *L*_*T*_ ≈ *L*_*M*_, a small or no increase in *L*_*T*_ is expected. Besides the increase in *L*_*T*_, adding a gain reduction is expected to result in broader masking patterns than in the standard condition, consistent with the idea that cochlear gain controls frequency selectivity ([37, 41]). For instance, a broadening of PTCs with gain reduction has been reported in a modeling study using the power-law temporal window model [37].

**Fig 5:**
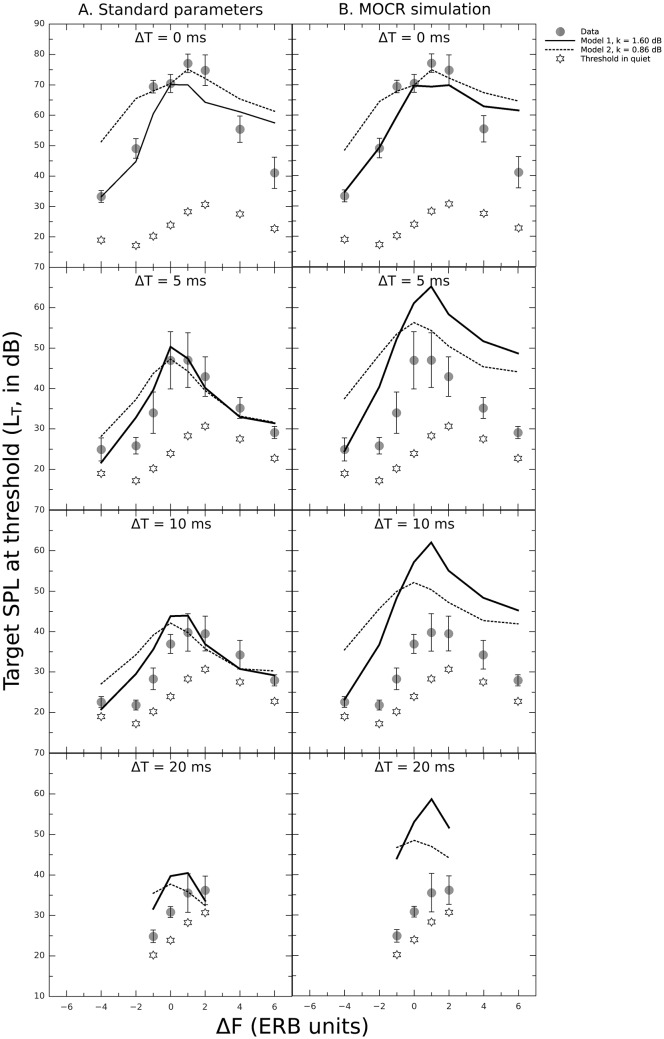
Measured (symbols) and simulated (lines) target SPL at threshold (*L*_*T*_, in dB) as a function of Δ*F* (in ERB units) for Δ*T* = 0–20 ms. Solid and straight lines show simulations using model 1 and model 2, respectively. The values of *k* used for each model are indicated in the legend. Stars indicate measured target SPLs in quiet. Error bars indicate ±1 standard deviation of the mean data. A: Simulations using standard model parameters. B: Simulations using a gain reduction of 15 dB to simulate the MOCR. The RMS errors between data and simulations are indicated in Table 1.

Simulated masking patterns for Δ*T* = 0–20 ms are presented in Fig 5 as solid (model 1) and dashed lines (model 2). Circles show the mean experimental data with ±1 standard deviation bars. RMS errors between data and simulations are presented in Table 1. The *k* values found to minimize the RMS error between data and simulations for the condition Δ*F* = 0, Δ*T* = 0 (step 1) were 1.6 and 0.86 dB for models 1 and 2, respectively. These values are within the range reported in [57] (*k* = 0.05–6 dB). As a reminder, all simulations in Fig 5 allowed for off-time and off-frequency listening. Preventing off-time and off-frequency listening in the models (i.e. fixing the center time of the temporal window at the start of the offset ramp of the target and the cochlear filter’s center frequency at *f*_*T*_) turned out to increase the total RMS error by about 4 dB for model 1 and 5 dB for model 2 in the standard configuration (results not shown). This suggests that listeners may have based their judgments on off-time and/or off-frequency listening cues in some conditions.

Consider first the results obtained using the standard model parameters (left column of Fig 5). Both models captured the pattern of results well in general, especially for Δ*T* = 5–20 ms (RMS error = 3.9 to 7.7 dB). For Δ*T* = 0, the predictions were less accurate (RMS error = 8.4 dB for model 1, 11.8 dB for model 2). To examine the strength of the relationship between models and data, the Pearson’s product-moment correlations were computed [69]. The values of *r*^2^, listed in Table 1, indicate that both models can mostly account for at least 60% of the variance. The lowest values of *r*^2^ obtained at Δ*T* = 10 ms are likely due to a floor effect on the lower frequency side (i.e. Δ*F*< 0). Accordingly, excluding the condition Δ*F* = -4 ERB units in the correlation analysis for Δ*T* = 10 ms resulted in *r*^2^ = 0.9 for model 1 and *r*^2^ = 0.8 for model 2. Overall, model 1 performed better than model 2 in all conditions. Both the DRNL filter and the power-law model are acknowledged models of nonlinear cochlear processing. Nonetheless, the parameters of the DRNL filter as used in model 1 have been adjusted to best explain nonlinear effects in temporal masking [52] and model 1 has been extensively tested on TF conditions. In contrast, model 2 has only been used to provide a qualitative description of TF masking data [37], which may explain its lower performance in predicting the present data.

**Fig 6:**
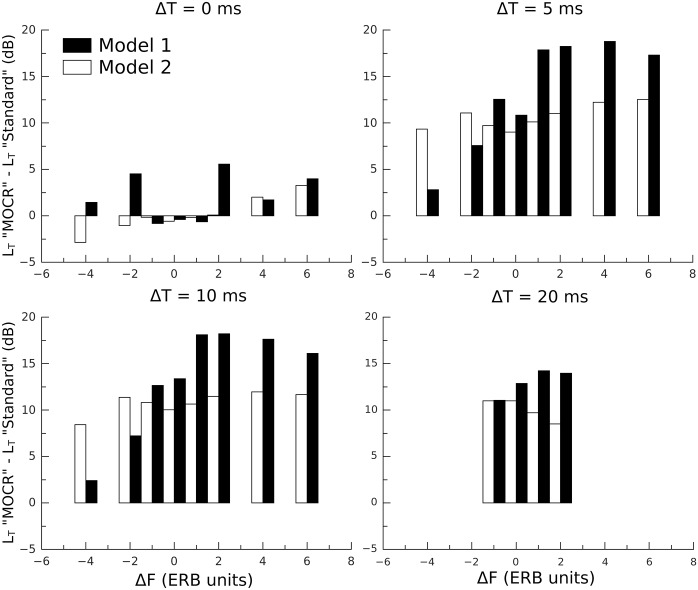
Difference in simulated *L*_*T*_ between conditions “MOCR simulation” and “standard” (in dB) as a function of Δ*F* (in ERB units) for Δ*T* = 0–10 ms. Filled bars show values for model 1. Empty bars show values for model 2.

Consider next the results obtained with MOCR simulation (right column of Fig 5). It can be seen that adding a gain reduction of 15 dB greatly decreased the prediction accuracy, increasing the total error by about 151% for model 1 and 71% for model 2. The values of *r*^2^, however, were hardly affected. This is likely due to the fact that adding a gain reduction mostly caused an increase in *L*_*T*_ at some Δ*F* s (see Fig 6), which did not grossly alter the shapes of the predicted masking patterns. Therefore correlations between models and data remained relatively strong. Fig 6 presents the difference between the *L*_*T*_ predicted by the “MOCR simulation” condition and that predicted by the “standard parameters” condition (in dB), as a function of Δ*F* for all Δ*T* s and both models. It can be seen that for both models, *L*_*T*_ hardly increased for Δ*T* = 0 and |Δ*F*|< 2. According to our hypothesis, this is supposed to happen when masker and target are similarly affected by the gain reduction. In these conditions, *L*_*M*_ and *L*_*T*_ at threshold were indeed in the same range (70–80 dB), i.e. both signals fell on the same portion of the compressive function. For Δ*T* = 0 and |Δ*F*| ≥ 2, the increase in *L*_*T*_ was ≤ 5 dB for both models. This is likely due to the fact that the two models, and in particular model 2, generally overestimated thresholds for Δ*T* = 0. Because the difference between *L*_*M*_ and *L*_*T*_ increases with increasing Δ*T* and Δ*F*, the effect of gain reduction should be greater for larger TF shifts. Accordingly, for Δ*T* > 0 and both models, *L*_*T*_ generally increased for all Δ*F* s, the increase being particularly large (up to 20 dB) for model 1 and Δ*F* ≥ 1 ERB unit.

Finally, to assess whether the simulated patterns broadened with MOCR simulation, *Q*_3_ were estimated for the simulated patterns in both conditions for Δ*T* = 0–10 ms (see Table 2). For both models and Δ*T* = 0–5 ms, the *Q*_3_ estimates slightly decreased with MOCR simulation. This indicates a mild broadening of the patterns when MOCR is simulated, consistent with [37, 41]. For Δ*T* = 10 ms, however, the differences in *Q*_3_ were negligible.

**Table 2:** *Q*_3_ estimates for the simulated masking patterns for Δ*T* = 0, 5, and 10 ms in both conditions.

	Δ*T* = 0	Δ*T* = 5	Δ*T* = 10
**model 1**			
standard	5.0	6.5	5.3
MOCR simulation	3.6	5.7	5.5
**model 2**			
standard	4.0	5.0	3.8
MOCR simulation	2.9	4.2	3.5

In summary, the results from the simulations in the standard condition showed that a well-established model of masking like the temporal window model is able to capture our pattern of TF masking data with Gaussian atoms reasonably well. This adds support to the interpretation that forward masking can be well described by combining cochlear nonlinearities with a temporal integrator [51]. Note that another implementation of the temporal window model that features a gain reduction stage, and that has also been tested on TF conditions, was proposed in [29]. To fit their data, the authors allowed *all* model parameters (i.e. those of the cochlear filter, gain reduction module, temporal integrator, and detection device) to freely vary. Because of the likely strong interaction between parameters, the choice of an adequate set of parameters for the gain reduction module to analyze the present data based on the results in [29] would have been critical. Thus, we did not test this model. Other masking models based on the assumption that forward masking results from adaptation in the auditory nerve (e.g. [70–72]) may also provide a good fit to our data. However, such models are usually based on complex signal detection mechanisms that may have complicated the comparison to the simple temporal window model approach. In addition, it is not intended in this study to compare the integration versus adaptation explanations of forward masking (for a discussion on this see e.g. [73]). Finally, unlike the temporal window model, adaptation models have not, to our knowledge, been tested on TF conditions.

The results from the simulations in the condition with MOCR simulation showed that reducing the cochlear gain in the model highly degraded the model’s ability to account for the present data. This supports the hypothesis that our TF masking data were not affected by gain-reduction mechanisms like the MOCR. This further suggests that models based on gain reduction are not well adapted to predict masking data involving Δ*T* s that are shorter than the MOCR onset delay (i.e. ≤ 25 ms). To predict masking data involving longer Δ*T* s, for instance previous TF masking data for long maskers, models based on gain reduction are presumably necessary. Although the temporal window model could well account for some masking data for long maskers (*D*_*M*_ > 100 ms) [52, 63], the present findings together with [29, 37] suggest that the prediction accuracy might be improved if a gain reduction stage were incorporated into the temporal window model. To examine this, our three-step simulation procedure was applied to the TF masking data reported in [18] using model 1, which performed better than model 2 in all conditions of Fig 5. In that study, masking patterns were measured for Δ*T* = 200, 300, 308, and 318 ms (note that these values of Δ*T* refer to the time delay between masker onset and target onset, while the values reported in [18] refer to the delay between masker offset and target offset) and Δ*F* = -2, -1.4, -0.7, 0, +0.8, +2.8, and +5 ERB units. The masker was a sinusoid with *D*_*M*_ = 300 ms, *F*_*M*_ = 4 kHz, and *L*_*M*_ = 70 dB. We selected this study because the values of *F*_*M*_ and *L*_*M*_ are comparable to ours. Moreover, the condition Δ*T* = 200 ms, Δ*F* = 0 (i.e. when the target was nearly temporally centered in the masker) is similar to our Δ*T* = 0, Δ*F* = 0 condition as a measure of intensity discrimination, which is required for the estimation of the optimum value of *k* (step 1). Given the large values of Δ*T* measured in [18], our hypothesis was that the MOCR configuration of the temporal window model would better predict these data than the standard configuration. Accordingly, the results (not shown) revealed that the model generally better captured the patterns of results in the MOCR than in the standard configuration. The simulations with gain reduction were better correlated with the data (total *r*^2^ = 0.73) than the simulations with standard parameters (total *r*^2^ = 0.34). However, because the thresholds were consistently overestimated in the MOCR condition, the RMS error between data and simulations tended to be slightly higher with MOCR (total RMS error = 14.7 dB) than without MOCR (total RMS error = 12.1 dB). The reasons for this overestimation are currently unclear.

Altogether, the results from the present simulations of TF masking for short and long maskers suggest that the spread of TF masking can be affected by the MOCR or other effects that reduce cochlear gain. By using spectrally and temporally maximally-compact stimuli, we avoided such effects in the present study and thereby obtained an estimate of the “basic” spread of masking for well-localized TF atoms.

### Implications for Audio Applications

To date, most audio applications exploit only spectral *or* temporal masking to predict masking in the TF domain. Spectral masking is usually modeled with the so-called “spreading function” of masking which, in its simplest form, is an asymmetric triangular function that approximates the general shape of masking patterns for narrowband maskers (e.g. [6, 8, 11, 22]). Temporal (forward) masking is usually modeled using a linear function of log(Δ*T*) (e.g. [7, 74, 75]). Some algorithms, though, exploit both spectral and temporal masking [9, 22–24] using a simple superposition of spectral and temporal masking functions. The linear combination of temporal and spectral masking to predict TF masking, however, has been invalidated in [16, 25]. Therefore, these TF masking models provide rather inaccurate predictions of TF masking. Noteworthy, in [75] the spectral and temporal masking functions are combined using a power-law function. This approach is more consistent with auditory processing than the linear combination, but the temporal masking function in [75] does not include the effect of Δ*F* on the temporal decay of masking. Thus, it is also no satisfactory TF masking model.

To obtain more accurate predictions of TF masking, audio applications might incorporate the present data, provided in S1 Dataset, for instance as a TF masking kernel (see e.g. [76, 77] for applications of the present data to sparse representations of audio signals). Alternatively, the temporal window model of masking could be used so long as short maskers are considered (unless a time-dependent gain reduction stage is implemented). A weakness of the temporal window model for audio applications, though, is its rather high computational load, especially when off-frequency and off-time listening are allowed for. In addition, the ability of the temporal window model to accurately predict TF masking for a range of masker frequencies and levels still needs to be evaluated.

Overall, combining the present data on the spread of TF masking for Gabor TF atoms with additional data on the frequency- and level-dependency of spectral masking for these atoms [25], and data on the additivity of simultaneous and non-simultaneous masking for multiple atoms [46, 68] constitutes a crucial basis for the development of a TF masking model adapted to atomic signal decompositions. Such a model might improve the estimation of TF masking in applications like audio coding, audio restoration, sparse approximation, or source separation, among others, as compared to presently available applications.

## Supporting Information

S1 Dataset Time-frequency masking function for a Gaussian TF atom with a center frequency of 4 kHz and a sensation level of 60 dB. The archive, compressed in a ZIP file (2.7 kB in size), includes the raw data in a MAT file, a Matlab/Octave function to interpolate the TF masking function at any sampling rate, and a “readme” text file.
(ZIP)
